# National Board of Medical Examiners Gives Second Examination

**Published:** 1917-04

**Authors:** 


### National Board of Medical Examiners Gives Second Examination

The second examination to be given by the National Board of Medical Examiners will be held in Washington, D. C., June 13, 1917. The examination will last about one week.

The following States will recognize the certificate of the National Board: Colorado, Delaware, Idaho, Iowa, Kentucky, Maryland, North Carolina, New Hampshire, North Dakota and Pennsylvania. Favorable legislation is now pending in twelve of the remaining States.

A successful applicant may enter the reserve corps of either the army or navy without further professional examination, if their examination papers are satisfactory to a board of examiners of these services.

The certificate of the National Board will be accepted as qualification for admittance into the graduate school of the University of Minnesota, including the Mayo Foundation.

Application blanks and further information may be obtained from the Secretary, Dr. J. S. Rodman, 2106 Walnut Street, Philadelphia.

